# Gonioscopy-assisted transluminal trabeculotomy in primary congenital glaucoma

**DOI:** 10.1016/j.ajoc.2022.101366

**Published:** 2022-01-29

**Authors:** Yunhe Song, Xiulan Zhang, Robert N. Weinreb

**Affiliations:** a State Key Laboratory of Ophthalmology, Zhongshan Ophthalmic Center, Sun Yat-sen University, Guangdong Provincial Key Laboratory of Ophthalmology and Visual Science, Guangdong Provincial Clinical Research Center for Ocular Diseases, Guangzhou 510060, China; b Shiley Eye Institute, Hamilton Glaucoma Center, Viterbi Family Department of Ophthalmology, University of California San Diego, La Jolla, CA, USA

**Keywords:** Primary congenital glaucoma, Childhood glaucoma, Gonioscopy-assisted transluminal trabeculotomy, GATT

## Abstract

**Purpose:**

To report a case of gonioscopy-assisted transluminal trabeculotomy (GATT) in a patient with primary congenital glaucoma.

**Observations:**

A three-year-old boy who presented with buphthalmos and elevated intraocular pressure. Despite the presence of iris and iris processes extending to Schwalbe's line, GATT was performed successfully.

**Conclusions:**

GATT may be successful with primary congenital glaucoma even when angle structures are not initially visible.

## 1 Introduction

Primary congenital glaucoma (PCG) is an unusual and inherited anomaly of the trabecular meshwork and anterior chamber angle.^1^ The intraocular pressure (IOP) is elevated with aqueous outflow obstruction and this eventually can lead to glaucomatous optic neuropathy and blindness.^1^ Trabeculotomy ab externo/interno, can achieve satisfactory IOP-lowering. With the advent of minimally invasive glaucoma surgeries (MIGS), gonioscopy-assisted transluminal trabeculotomy (GATT) also is effective in treatment of PCG patients.^2^^,^^3^ With visible angle structures, Schlemm's canal is observed and its inner wall is incised to increase outflow and lower the IOP.

Here, we report the successful use of GATT in a PCG patient in whom the angle structures were not initially visible.

## 2 Case report

A 3-year-old young boy, full-term, was referred with a 3-month history of enlargement of both globes and photophobia. His visual acuity was 0.2 in the right eye (OD) and 0.04 in the left eye (OS), respectively. He had no previous ophthalmic history and no family history of ocular disorders or systematic abnormalities.

**Fig. 1:**
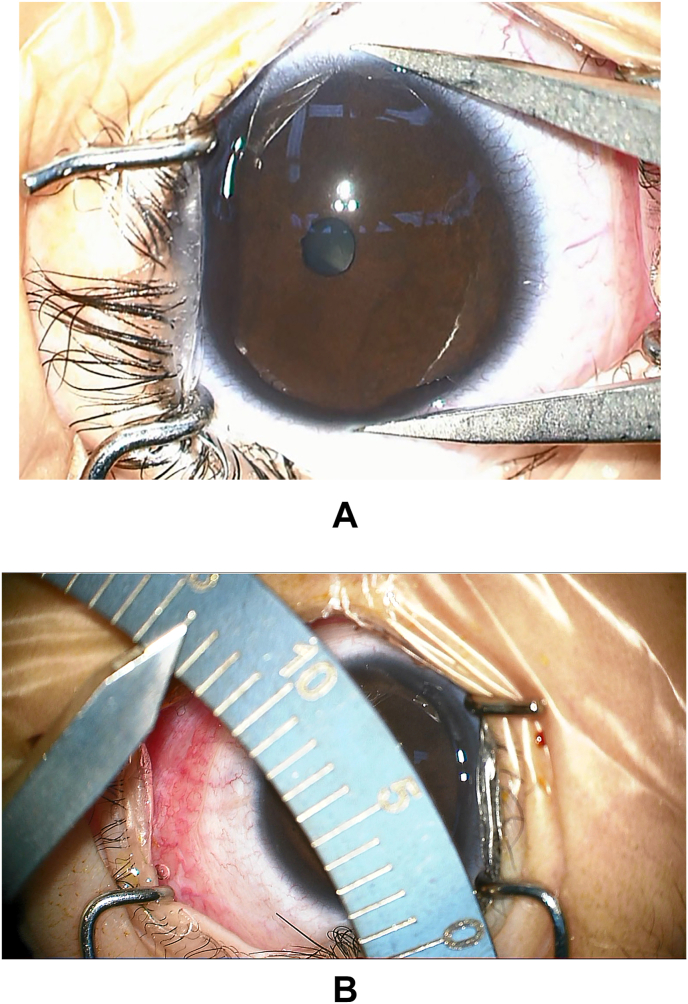
The diameter of the cornea is 14 mm in both eyes.

Under general anesthesia, corneas were enlarged (14 mm), but clear (Fig. 1) and there were no Haab's striae in either eye. In both eyes (OU), the central and peripheral anterior chamber depths were deep. With rebound tonometry iCare (iCare USA, Raleigh, NC), IOP was measured as 27 mm Hg OD and 34 mm Hg OS. Direct ophthalmoscopy revealed deep cupping of the right optic disc (cup-to-disc ratio of 0.6). The pupil OS did not dilate and the fundus could not be visualized.

**Fig. 2:**
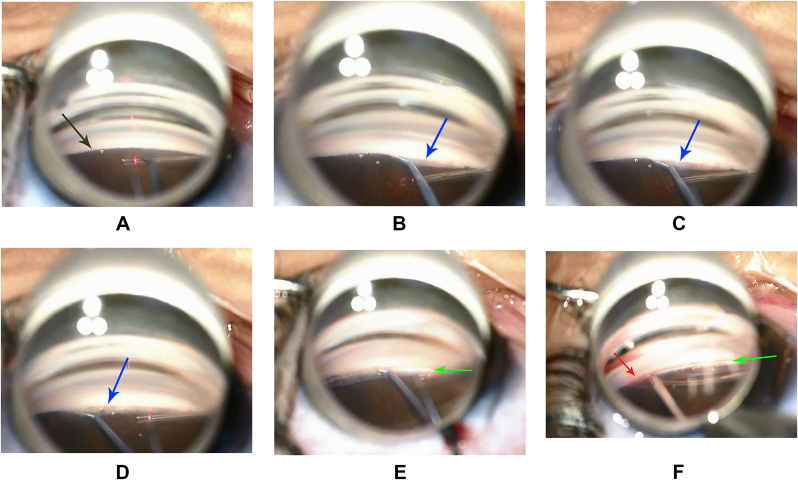
**The key procedures of operation in the right eyes.** **A.** Under gonioscopy, the iris covered the entire angle without visibility of any other angle structures (black arrow). **B-D.** Separation of the iris root and revealing of angle structures with clear trabecular meshwork (blue arrow). **E.** Dissection of the inner wall of Schlemm's canal (green arrow). **F.** Insertion the iTrack into the Schlemm's canal (red arrow, green arrow indicates the dissected Schlemm canal). iTrack diameter: 250 μm. (For interpretation of the references to colour in this figure legend, the reader is referred to the Web version of this article.)

He was diagnosed with PCG OU and scheduled for GATT. At the time of surgery, the anterior chambers were deepened with viscoelastic. With gonioscopy, the iris was observed to cover the entire angle up to Schwalbe's line (Fig. 2A), and angle structures were not visible OD. Next, a hook was used to gently strip the iris from the angle and angle structures were visualized (Fig. 2B–D). GATT, as described previously,^2^ was performed without intraoperative complications. (Fig. 2E–F) (**See surgical video 1**). In brief, the microcatheter was inserted into the Schlemm's canal and passed circumferentially around the entire canal. The two ends of the microcatheter were fixed and retrieved within the chamber using microsurgical forceps. The blinking head of the catheter allows for the visualization of the actual dynamic positioning and avoid misleading around the angle.

Supplementary video related to this article can be found at https://doi.org/10.1016/j.ajoc.2022.101366.

The following is the supplementary data related to this article:

On the first post-operative day, IOP was 10 mm Hg OD. After one week, his IOP was 6-mm Hg. The same surgery was performed one week later in OS with similar results. GATT was successfully performed with twice 270-degree goniotomy (**See surgical video 2**).

Supplementary video related to this article can be found at https://doi.org/10.1016/j.ajoc.2022.101366.

**Fig. 3:**
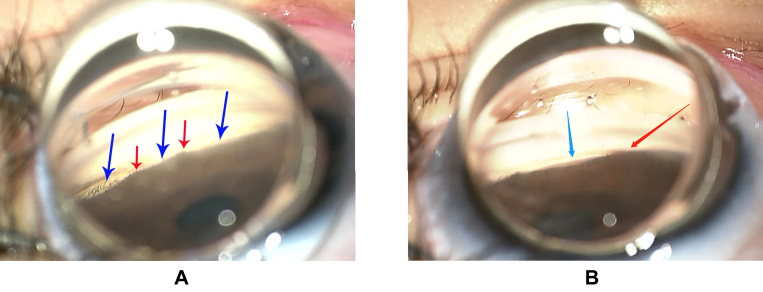
**Post-operative images of gonioscopy**-**assisted transluminal trabeculotomy.** A. This is the gonioscopic image of right eye following six months post-operatively. The dark blue arrows indicated the trabecular shelf stays (the dissected Schlemm's canal of goniotomy) and the red arrows showed the peripheral anterior synechia formation. B. This is the gonioscopic image of left eye after six months post-operatively. The light blue arrow clearly indicated the trabecular shelf stays (the dissected Schlemm's canal of goniotomy). The red arrow showed the peripheral anterior synechia formation. (For interpretation of the references to colour in this figure legend, the reader is referred to the Web version of this article.)

The following is the supplementary data related to this article:

Subsequent follow-up visits over 6 months follow-up showed a consistent reduction OU of IOP. The post-operative gonioscopic images were shown in Fig. 3 bilaterally, in which the dissected Schlemm's canal and localized cleft were presented clearly. And there are several small parts of peripheral anterior synechia and cleft closure at 6-month follow-up.

## 3 Discussion

GATT was first proved to be successful in adult glaucoma^4^ and have played its role in juvenile and childhood glaucoma.^2^^,^^5^ This patient with PCG had unique angle structures as the iris root covered the entire angle. Neither the scleral spur nor Schwalbe's line were completely visible with gonioscopy. By recognizing and then enhancing the visibility of the anatomical structures by locating the Schlemm's canal precisely, GATT could be performed successfully (**See surgical video 3**).

Supplementary video related to this article can be found at https://doi.org/10.1016/j.ajoc.2022.101366.

The following is the supplementary data related to this article:

The complications of GATT should be considered. The iridodialysis, post-operative IOP spike, and hyphema are common post-operative complications.^6^ The potential mechanisms of the above complications are the dissection of Schlemm's canal and communication of anterior chamber and suprachoroidal space. The GATT could also be done with 5-0 prolene suture, while by microcatheter would ensure a right position with a blinking head. The precise surgical technique is the key to success.

## Funding

No funding or grant support.

## Authorship

All authors attest that they meet the current ICMJE criteria for Authorship.

## Patient consent

Written consent to publish this case has been obtained. This report does not contain any personal identifying information.

## Declaration of competing interest

The following authors have no financial disclosures: YHS, XLZ and RNW.
